# Natural AMPK Activators in Cardiovascular Disease Prevention

**DOI:** 10.3389/fphar.2021.738420

**Published:** 2022-01-03

**Authors:** Reza Heidary Moghaddam, Zeinab Samimi, Sedigheh Asgary, Pantea Mohammadi, Soroush Hozeifi, Fatemeh Hoseinzadeh‐Chahkandak, Suowen Xu, Mohammad Hosein Farzaei

**Affiliations:** ^1^ Clinical Research Development Center, Imam Ali and Taleghani Hospital, Kermanshah University of Medical Sciences, Kermanshah, Iran; ^2^ Pharmaceutical Sciences Research Center, Health Institute, Kermanshah University of Medical Sciences, Kermanshah, Iran; ^3^ Isfahan Cardiovascular Research Center, Cardiovascular Research Institute,.Isfahan University of Medical Sciences, Isfahan, Iran; ^4^ Medical Biology Research Center, Kermanshah University of Medical Sciences, Kermanshah, Iran; ^5^ School of Medicine, Birjand University of Medical Sciences, Birjand, Iran; ^6^ Social Determinants of Health Research Center, Birjand University of Medical Sciences, Birjand, Iran; ^7^ Department of Endocrinology and Metabolism, The First Affiliated Hospital of USTC, Division of Life Sciences and Medicine, University of Science and Technology of China, Hefei, China; ^8^ Medical Technology Research Center, Health Technology Institute, Kermanshah University of Medical Sciences, Kermanshah, Iran

**Keywords:** atherosclerosis, cardiovscular disease, AMPK, natural products, drug discovery

## Abstract

Cardiovascular diseases (CVD), as a life-threatening global disease, is receiving worldwide attention. Seeking novel therapeutic strategies and agents is of utmost importance to curb CVD. AMP-activated protein kinase (AMPK) activators derived from natural products are promising agents for cardiovascular drug development owning to regulatory effects on physiological processes and diverse cardiometabolic disorders. In the past decade, different therapeutic agents from natural products and herbal medicines have been explored as good templates of AMPK activators. Hereby, we overviewed the role of AMPK signaling in the cardiovascular system, as well as evidence implicating AMPK activators as potential therapeutic tools. In the present review, efforts have been made to compile and update relevant information from both preclinical and clinical studies, which investigated the role of natural products as AMPK activators in cardiovascular therapeutics.

## 1 Introduction

Cardiovascular diseases (CVD) in most countries are now one of the top concerns of the health care system, and according to a disease statistics report released by the World Health Organization (WHO) in 2019, about 17.9 million people died from CVD (WHO 2021). It is estimated that with the increased prevalence of CVD, the number of expected deaths due to CVD will increase to 24 million per year by 2030. Therefore, the increase in high-risk patients prone to CVD, as well as the high cost of treatment and subsequent debilitating complications, forewarn us of regarding the need for intensive investigation into the pathogenesis of CVD (Afzal 2021).

One of the therapeutic targets for CVD is the AMP-activated protein kinase (AMPK) which is found in most mammalian tissues such as cardiovascular organs (Xu and Si, 2010). In the mid-1990s, Lopaschuck’s Laboratory first investigated the role of AMPK in the heart (Kudo et al., 1995, Kudo et al., 1996). The modifiable major risk factors that lead to CVD are excess weight, dyslipidemia, increased blood pressure, diabetes, and metabolic syndrome, in which AMPK activators may confer benefits (Nellaiappan et al., 2019). Mechanistic research has now discovered signaling pathways that link AMPK to CVD. These routes seem to be interconnected and can be considered as new goals for the design and development of treatment strategies in the future. On the other hand, AMPK deregulation is thought to be related to various cardiovascular disorders (Costantino, Paneni, and Cosentino 2016). Activated AMPK inhibits energy-consuming process and stimulates several metabolic pathways for energy-production and cell survival. AMPK activation in response to cellular energy stress occurs due to the reduction in the ATP/AMP ratio in the cytosol (Hardie et al., 2012). In fact, AMPK is a heterotrimeric serine-threonine kinase, which acts as a metabolic sensor to coordinate catabolic and anabolic pathways in the heart (Arad et al., 2007). In recent years, researchers reported various non-pharmacological and natural compounds based therapeutics from animal studies and considered them in the CVD treatment. Some of natural products have shown promising *in vitro* and *in vivo* activities in the AMPK regulation. In this review, we present an overview of mechanisms regulating AMPK in the cardiovascular system; highlighting the role of AMPK signaling in CVD, followed by a description of natural AMPK activators as potential therapeutic tools.

## 2 AMPK: Upstream and Downstream Signaling Pathways

Maintaining energy homeostasis is a fundamental biological pathway that occurs within all living cells. AMPK is a key gatekeeper of energy homeostasis that is activated in response to different stimuli, leading to intracellular decrease in ATP levels. Actually, under cellular stress and/or energy stress, AMPK is activated in response to decreased ATP production or increased ATP consumption (Mihaylova and Shaw, 2011). AMPK exists as a heterotrimer, containing a catalytic subunit (α), and two regulatory subunits (β and γ) (Zhang et al., 2013). Each subunit has multiple isoforms (α1, α2, β1, β2, γ1, γ2, γ3), giving rise to a total of 12 feasible heterotrimer combinations. The kinase activity of α subunit is stimulated more than 100-fold by phosphorylation of a conserved threonine residue located on the kinase activation loop (Thr 172 in α1/α2). The main upstream kinase that is responsible for the phosphorylation of this site was detected as a heterotrimeric complex comprising tumor suppressor kinase LKB1, mouse protein 25 (MO25), and sterile-20-related adaptor (STRAD) (Hardie, 2014). Subsequently, the Ca^2+^/calmodulin-dependent protein kinase kinases (CaMKKs) (especially CaMKKβ) were discovered as an alternate enzyme playing role in Thr172 phosphorylation in response to the rise in intracellular Ca^2+^ (Nguyen et al., 2016). Additional studies have proposed that TAK1/MAP3K7 (Transforming growth factor beta-activated kinase 1)/(Mitogen-activated protein kinase 7), a MAPKKK family member, may also phosphorylate Thr172 (Tamargo-Gomez and Marino, 2018). Under conditions of decreased intracellular ATP concentrations, binding of AMP and/or ADP to the γ-regulatory subunit activate this kinase by three complementary mechanisms: promotes phosphorylation of AMPK by the upstream kinases, protects the enzyme against dephosphorylation through conformational changes that inhibits protein phosphatases, as well as causes allosteric activation which is only limited to AMP (Hardie, 2014).

### 2.1 Coordinated Regulation of Growth and Autophagy by AMPK

Under conditions of nutrient stress*,* metabolic checkpoint protein*s* like AMPK, can inhibit cellular growth. AMPK suppresses mammalian target of rapamycin complex 1 (mTORC1)*.* mTORC1 comprised of the three core subunits (mTOR, Raptor, and mLST8) is a key regulator of ribosomal biogenesis and protein synthesis (Bond, 2016). AMPK controls the mTORC1 through phosphorylation/inactivation of the tumor suppressor TSC2. In addition, findings obtained from lower eukaryotes with TSC2 depletion or TSC2^−/−^ mouse embryonic fibroblasts (MEFs) demonstrated that AMPK can also inhibit mTORC1 pathway through direct phosphorylation of Raptor (Mihaylova and Shaw, 2011). In addition to the control of cell growth, mTORC1 also regulates autophagy, lysosomal degradation of intracellular components sequestered within autophagosome to supply sufficient metabolites in low availability of nutrients or to increase energy demands (Papinski and Kraft, 2016). The process by which TORC1 negatively controls the autophagic machinery was first defined in the yeast. Genetic evaluations of autophagy-deficient mutants result in identifying >30 essential autophagy-related genes (Atg34 and Atg81). The main target is a collection of three proteins including the serine/threonine kinase Atg1and its accessory proteins Atg13 and Atg17. mTORC1 can directly suppress this complex in a nutrient-dependent manner (Alers et al., 2012). Phosphorylation of the Ulk1 (and Ulk2), Atg1 mammalian homologs, moreover their regulatory subunits probably is the mechanism by which mTORC1 can inhibit autophagy in mammals. In addition, AMPK also has the ability to directly phosphorylate Ulk1 complex and induced autophagy induction as well as maintain mitochondrial homeostasis control. Based on the literature, tumor suppressor p53 and the cyclin dependent kinase inhibitor p27 are recruited as other targets of AMPK in growth control (Mihaylova and Shaw, 2011).

AMPK can be regulated by metabolic factors, such as leptin and adiponectin, two well-known adipokines secreted by adipocytes. Through AMPK activation, both hormones inhibit the activity of acetyl coenzyme A carboxylase (ACC) and 3-hydroxy-3-methylgutaryl-coenzyme A (HMG-CoA) reductase, which are responsible for cholesterol and fatty acid synthesis in skeletal muscle and other metabolic tissues (Hardie, 2004; Wang et al., 2018). In addition, hormone sensitive lipase (HSL) and adipocyte triglyceride lipase (ATGL) are the other substrates in adipose tissues which are activated by AMPK (Mihaylova and Shaw, 2011).

Exercise and insulin induce glucose uptake into skeletal muscle through various signaling pathways. Both exercise and insulin can trigger GLUT4 translocation from intracellular vesicles to the cell surface. AS160 and TBC1D1 are two RabGAPs, which have been implicated in this process. AS160 highly expressed in heart, oxidative muscles, and white adipose tissue (WAT). On the other hand, its homologue, TBC1D1 mostly expressed in skeletal muscle, involves exercise-mediated GLUT4 trafficking. RabGAPs control the activity of Rab GTPases, which have been implicated in eukaryotic vesicular trafficking (Stockli et al., 2015). AMPK plays a role in glucose uptake via effects on the AS160 and TBC1D1 (Mihaylova and Shaw 2011).

In addition to the rise in energy expenditure via inducing fatty acid and glucose oxidation, AMPK also seems to control mammalian energy intake through effect on the regions in hypothalamus. For example, AMPK is suppressed in the condition of elevated levels of glucose and insulin, whereas it is stimulated by ghrelin (a gut hormone increasing appetite and food intake) (Hardie, 2004). Generally, AMPK plays a role in the downregulation and upregulation of biosynthetic and catabolic pathways, respectively by acute regulation of the metabolic enzymes and transcriptional changes (Hardie, 2004).

### 2.2 Effect of AMPK in Metabolic Regulation via Impacting Transcription and Metabolic Enzymes

AMPK has been recognized as a key regulator of some transcription factors, co-activators, the histone acetyltransferase p300, histone deacetylase family, and histones themselves. For example, it was reported that AMPK, through phosphorylation of histone H2B, upregulates stress related genes including p21 and cpt1c (Mihaylova and Shaw, 2011). It has recently been proved that AMPK activation affects circadian clock regulation. Mammalian circadian clocks require activators and repressors that regularly control transcription. CLOCK and BMAL1 act as potent stimulators of the expression of Period (Per1, Per2, and Per3) and Cryptochrome genes (Cry1 and Cry2), the encoded proteins of which also inhibit CLOCK: BMAL1. Cry1/2 are targeted for ubiquitination and degradation by ubiquitin ligase complex SCF (SKP1-CUL1-F-box protein) (Huber et al., 2016). AMPK, via phosphorylation of the Cry1, leads to direct binding of the Fbox protein Fbxl3 to Cry1. It has been demonstrated that AMPK can also control the circadian clock by phosphorylation of Casein kinase (Mihaylova and Shaw, 2011).

AMPK also phosphorylates and inactivates the lipogenic transcriptional factors such as carbohydrate-responsive element-binding protein (ChREBP) and sterol regulatory element binding protein -1c (SREBP-1c). These two regulators dictate the expression of major lipogenic enzymes including acetyl-CoA carboxylase (ACC), ATP-citrate lyase (ACLY), acetyl-CoA synthetase (ACS), fatty acid synthase (FAS), stearoyl-CoA desaturase-1 (SCD1), and glycerol-3- phosphate acyltransferase (GPAT) that promote lipogenesis in the liver (Xu et al., 2013). Furthermore, there are observations that phosphorylation of histone acetyltransferase p300 by AMPK can also affect the acetylation and activity of ChREBP (Mihaylova and Shaw, 2011).

Reduction of cellular energy levels during prolonged fasting or intense exercise results in AMPK stimulation and prevention of hepatic gluconeogenesis to some extent through phosphorylation and inactivation of CREB-regulated transcriptional coactivator 2 (CRTC-2) (Altarejos and Montminy, 2011). AMPK also reduces gluconeogenesis through class IIa family of histone deacetylases (IIa HDACs) inactivation leading to reduced de-acetylation and activation of FOXO transcription factor in liver (Mihaylova and Shaw, 2011). It was demonstrated that CRTC-1 is a direct AMPK target interacting with the CREB homologue-1 (CRH-1) transcription factor *in vivo*. Activating AMPK through reduction of CRTC-1 and CRH-1 activity is responsible for lifespan extension (Mair et al., 2011). Some findings also indicated the involvement of AMPK in the phosphorylation of nuclear receptors HNF4α (NR2A1) and TR4 (NR2C2), zinc-finger protein 692 (ZNF692), and the co-activator PGC-1α. More studies are required to identify exact roles of these events (Mihaylova and Shaw, 2011). It was also reported that AMPK operates in accordance with another metabolic sensor, the NAD^+^-dependent type III deacetylase SIRT1, thereby regulating the expression of genes responsible for cellular energy metabolism in metabolic tissues. AMPK promotes SIRT1 activity by increment of cellular NAD^+^ levels, leading to fatty acid oxidation and mitochondrial gene expression. SIRT1 regulates the activity of transcription factors and coregulators including forkhead box class O (FOXO) transcription factors, peroxisome proliferator-activated receptor-gamma (PPARγ), and p53 (Canto et al., 2009).

### 2.3 Regulation of Cell Polarity, Migration, and Cytoskeletal Dynamics

In addition to many findings reporting AMPK’s role in cell growth and metabolism, some studies have documented that LKB1 and AMPK play critical roles in cell polarity from invertebrates to mammals. For instance, certain epithelial cell polarization in mammalian cell culture and polarity of the cells during critical asymmetric cell divisions in *Drosophila* are attributed to LKB1 and his orthologs, respectively (Mihaylova and Shaw 2011; Mirouse and Billaud 2011). There are also several studies reporting the role of AMPK in cell polarity. For example, in mammalian MDCK cells, AMPK leads to suitable repolarization and tight junction assembly after calcium switch. Myosin II was recognized as a main downstream target of AMPK. In *Drosophila* embryo, Myosin II was activated through phosphorylation of its regulatory light chain (referred as MRLCII). However, MRLCII is not targeted as direct substrate of AMPK and it thus becomes important to identify the exact signaling mechanism (Mihaylova and Shaw 2011; Mirouse and Billaud 2011). Besides, CLIP-170 (CLIP1), the microtubule plus end protein, is another substrate of AMPK that affects microtubule assembly (Mihaylova and Shaw 2011).

## 3 Role of AMPK Signaling in Cardiovascular Diseases

AMPK is implicated in the regulation of cardiovascular function via coordinating several critical physiological and pathological cellular pathways in the cardiovascular system, which includes both metabolic as well as non-metabolic components of different cell types such as vascular cells, fibroblasts and cardiomyocytes. In the following section, we briefly summarize the important role of AMPK in CVD.

### 3.1 Atherosclerosis and Ischemic Stroke

Atherosclerosis is considered as a known chronic inflammatory reaction of artery walls caused by lesions and plaques. Eventually, thrombus formation on atherosclerotic plaques leads to heart attacks and ischemic stroke (Libby et al., 2009; Xu et al, 2021). This disease is related to both increases in low-density lipoprotein cholesterol (LDL-C) and chronic low-grade inflammation, both of which are regulated by AMPK. AMPK regulates proliferation, apoptosis, migration, and autophagy of smooth muscle cells and endothelial cells as well as macrophages. AMPK reduces atherosclerosis progression by inhibition of cell proliferation (*via* p53 and mTOR) and induction of autophagy (Wang et al., 2017). AMPK arrests cell cycle progression by increasing cells in G0/G1-phase and decreasing cells in S- and G2/M-phase (Igata et al., 2005).

Furthermore, AMPK influences the level of adiponectin, which decreases vascular smooth muscle cell migration induced by insulin-like growth factor-1 (Motobayashi et al., 2009). AMPK affects the antioxidant condition of endothelial cells (Colombo and Moncada 2009) and *in vivo*, a decrease in AMPK will eventually increases atherosclerosis and ER stress (Dong et al., 2010). Interestingly, AMPK inhibits the macrophage proliferation induced by LDL and atherosclerosis (Ishii et al., 2009).

### 3.2 Hypertension

Hypertension is one of the main risk factors for CVD besides elevated LDL-C and inflammation. The changes in the structure of vessels commonly seen in hypertension is due to vessel remodeling and/or hypertrophy. In hypertensive rats, AMPK is overexpressed as revealed by cDNA microarray (Kurdi et al., 2004). Metformin can activate AMPK and inhibit NF-kB, thus attenuating the expression of adhesion molecules and pro-inflammatory genes induced by cytokines (Hattori et al., 2006), which are crucial for progression of hypertension (Zhou et al., 2010). Indeed, in insulin-sensitive tissues from hypertensive rats, impaired adiponectin-AMPK pathway has been observed (Rodríguez et al., 2008). AMPK causes vasodilation via elevating endothelial nitric oxide by promoting eNOS phosphorylation (Chen et al., 1999), angiotensin-converting enzyme 2 (Zhang et al., 2018), and elevating calcium levels (Schneider et al., 2015).

### 3.3 Ischemia-Reperfusion (I/R) Injury

A high amount of energy generated by the oxidation of different substrates like fatty acids and glucose, is needed for heart to function normally (Rösen et al., 1984; Lloyd et al., 2004). AMPK regulates energetic homeostasis through glycolysis and glucose uptake stimulation, meanwhile attenuating energy consumption (Hue et al., 2002; Dyck and Lopaschuk 2006). AMPK is stimulated throughout low-energy cellular conditions, like myocardial ischemia. Myocardial I/R damage (oxidative and inflammatory damage to the cardiac muscle due to reperfusion after ease of ischemia), is a vital cardiopathic process in which AMPK plays a major role (Gr and Be 2009).

AMPK preserves ischaemic cardiomyocytes, via various processes. On the one hand, AMPK promotes the translocation of glucose transporter type 4 (GLUT4) to the sarcolemmal membrane and boost glucose uptake (Russell et al., 1999). On the other hand, when oxygen supply is restored during reperfusion, extra AMPK stimulation elevates oxidation of fatty acid (Kudo et al., 1995). The effect of AMPK on fatty acid oxidation brings the protective role of AMPK during early reperfusion into question (Dyck and Lopaschuk 2006). Additionally, following the mitochondrial damage in the course of reperfusion, mitochondrial respiratory capacity is maintained by AMPK via opening of the mitochondrial permeability transition pore (mPTP) (Qi and Young 2015).

Interestingly, AMPK was later described as a remarkable cardiac savior against cardiomyocyte apoptosis (Kewalramani et al., 2009). Moreover, in ischaemic heart, AMPK is a vital regulator of autophagy. Autophagy is a survival process to save the substances and energy demand in ischaemic myocardium (Yan et al., 2005; Hamacher-Brady et al., 2007; Nakai et al., 2007; Rothermel and Hill 2007).

Endothelial AMPK might be an important factor in tissues exposed to ischaemic stress. Eventually, It was shown that AMPK is involved in adiponectin mediated pro-angiogenic function (Ouchi et al., 2004). Besides, vascular endothelial growth factor (VEGF) mRNA stability was extended by glucose deprivation (Yun et al., 2005). Metformin administration before, throughout, or following myocardial ischemia has been proved to inhibit ischemia-reperfusion damage and associated adverse left ventricular remodeling (El Messaoudi et al., 2011). In addition, AMPK is neuroprotective against ischaemic stroke (Culmsee et al., 2001; Dasgupta and Milbrandt 2007; Kuramoto et al., 2007).

### 3.4 Left Ventricular Remodeling After Myocardial Infarction

Left ventricular (LV) remodeling always occur because of cardiac damage after I/R. Cardiac fibrosis, the extracellular matrix (ECM) accumulation in the myocardium, is classified as either reactive interstitial fibrosis or reparative fibrosis, defined by the pathophysiological status (Travers et al., 2016). The inflammatory responses will start quickly after MI and trigger fibrogenesis, leading to the clearance and replacement of damaged cardiac fibroblasts in the injured necrotic part by a solid fibrotic scar (Weber et al., 2013; Daskalopoulos et al., 2014). This is vital and protective for wound healing and viability *via* preserving myocardial integrity and prevent cardiac rupture development. Within the infarcted area of AMPKα1-deficient mouse hearts, collagen cross-linking and myodifferentiation are remarkably decreased, causing faulty scar collagen maturation, compromised scar contractility, and worsening LV dilatation 30 days post-MI (Noppe et al., 2014). Later, fibrosis will extends to the non-infarcted myocardium, creating myocardial stiffness, diastolic dysfunction, systolic dysfunction, HF and eventual death (Weber et al., 1989; Masci et al., 2014).

Immunosuppressive and anti-inflammatory functions of AMPK have been shown in different cell types and autoimmune/inflammatory diseases (Salt and Palmer 2012). Metformin administration could cause acute AMPK activation (Soraya et al., 2012) and chronic AMPK pre-activation (Soraya et al., 2015) which in turn decreases cardiac remodeling through attenuating infiltration of peripheral neutrophils into the myocardial tissue after MI. On the other hand, during cardiac inflammatory reaction, tumor necrosis factor-α (TNF-α), a pro-inflammatory cytokine, was increased. AMPK also works to oppose cardiomyocyte necrosis induced by TNF-α and as well as inflammatory cell infiltration into the ischaemic myocardia (Peng et al., 2009).

Reactive oxygen species (ROS) is a key player in cardiac fibrosis which can be prevented by ROS scavenger treatments (Richter and Kietzmann 2016). SIRT proteins, a group of class III histone deacetylases that play a role in metabolism, decrease cardiac injury/fibrosis induced by ROS via positive feedback loop involving AMPK (Chong et al., 2012). Liu and coworkers has recently demonstrated the effects of anti-inflammatory and anti-oxidative effects of Arctigenin as a natural lignan compound. They found that Arctigenin decreased apoptosis of cardiomyocytes *via* AMPK/SIRT1 pathway in myocardial I/R Injury (Liu et al., 2020). Interestingly, SIRT3 (Lombard and Zwaans 2014) and SIRT6 (Wang et al., 2016) lower oxidative stress via an AMPK-related pathway as well.

### 3.5 Cardiac Hypertrophy

Cardiac hypertrophy, one of the main pathological mechanisms leading to cardiac remodeling. It is described as an increase in gene transcription and protein translation, increased size of cardiomyocytes, and a greater extent of sarcomere organization. Physiological cardiac hypertrophy occurs due to a physiological situation as a compensatory reaction to intense exercise in many athletes. However, pathological hypertrophy occur in response to myocardium mechanical stress, such as volume/pressure overload observed in hypertension or valvular heart disease or myocardial ischemia. In the case of chronic hypertension, pathological hypertrophy turns into abnormal remodeling and dysfunction that eventually promotes heart failure (HF) development (Frey and Olson 2003). Several groups focused on the study of AMPK as a pharmacological target against cardiac hypertrophy and subsequently HF. For instance, an experimental research was conducted to investigate the anti-hypertrophic effect of QF84139 as a novel small molecule (synthesized pyrazine derivative) in phenylephrine-induced hypertrophic model. One notable finding in this study is that QF84139 acts as an effective AMPK activator for the treatment of cardiac hypertrophy (Li et al., 2021). In another recent study (Zhang et al., 2021), Zhang et la have revealed that Bawei Chenxiang Wan as a Tibetan herbal medicine that prevents cardiac hypertrophy in isoprenaline-induced rats by activating AMPK/PPAR-α signaling. AMPK as a key player in metabolic pathways, has a vital function in various cellular processes to protect against cardiac hypertrophy, through regulating energy supply, protein synthesis, autophagy, cytoskeletal network expansion, transcription, ER stress, and microRNA expression (Frey and Olson 2003; Horman et al., 2012).

#### 3.5.1 Energy Supply

Cardiac metabolism disturbances is an important factor of hypertrophic process to meed the elevated energy requirements due to increased cardiac workload. Deregulation of cardiac energetics and cardiac dysfunction afterwards could occur as a result of changes in the metabolic status of the cardiomyocytes. AMPK is a key regulator in metabolic processes of the cardiomyocytes (Nascimben et al., 2004; Horman et al., 2012). Therefore AMPK has gained intensive attention as a target in anti-hypertrophic research plans.

#### 3.5.2 Protein Synthesis

An increase in the size of cardiomyocytes and protein synthesis, which cause a thickening of the LV walls (concentric hypertrophy), is the most prominent feature of hypertrophy. AMPK can efficiently inhibit two major mechanism governing protein synthesis. The first one is the eukaryotic elongation factor-2 (eEF2) kinase/eEF2 pathway (Chan et al., 2004) and the other one is (mTOR)/p70 ribosomal protein S6 kinase (p70S6K) axis, the Akt/mammalian target of rapamycin (Zarrinpashneh et al., 2008; Fu et al., 2011). Mice lacking AMPKα2 are largely affected by LV hypertrophy following pressure overload (Zarrinpashneh et al., 2008) or shortly after aortic banding (Zhang et al., 2008). In line with previous observations, AMPK is capable of suppressing cardiac hypertrophy after transverse aortic banding (Li et al., 2014). Moreover, in hypertrophic hearts, AMPK interacts with protein quality control mechanism by promoting the clearance of malfunctioning mitochondria (Li et al., 2015).

#### 3.5.3 Cytoskeleton

Another characteristic of cardiomyocyte hypertrophy is cytoskeletal network expansion, in particular microtubule densiccation, contributing to cardiac contractile dysfunction. AMPKα2 phosphorylates MAP4 (Microtubule-associated protein 4), leading to the decrease in microtubules accumulation and densiccation (Fassett et al., 2013).

#### 3.5.4 Transcription Regulation

AMPK accomplishes its anti-hypertrophic properties *via* another fascinating mechanism of transcriptional regulation via NFAT pathway (Chan et al., 2008). AMPK inhibit cardiomyocyte hypertrophy, via preventing NFAT translocation to the nucleus. There are also other key regulators involved such as c-myc, the FoxO1/muscle RING-finger protein-1(FoxO1/MuRF1) pathway and the extracellular signal-regulated kinases (ERK) (Esposito et al., 2001; Shibata et al., 2004) and MuRF1 (Arya et al., 2004; Chen et al., 2010). Mitochondria metabolic functions have been shown to be regulated by collaborative actions of c-myc and AMPK (Edmunds et al., 2015).

#### 3.5.5 Autophagy Induction

Autophagy is a remarkable mechanism by which the clearance of unwanted cellular parts and recycles occur (Fimia et al., 2013; Reggiori and Klionsky 2013). Lately, it has been suggested that autophagy has dual roles in cardiac hypertrophy. Constitutive autophagy preserves cardiac function/structure, however extra autophagy activation promotes autophagic cell death and eventually provokes cardiac hypertrophy (Li et al., 2015). Through phosphorylation of the stimulator of autophagy-ULK1, AMPK can promote cardiomyocyte autophagy (Herrero-Martín et al., 2009; Kim et al., 2011).

#### 3.5.6 ER Stress Attenuation

ER stress is defined as an ER homeostasis imbalance and ER dysfunction. Extra ER stress leads to unfolded/misfolded proteins accumulation and subsequently to cardiac hypertrophy by triggering cell death (Kaufman, 2002; Wang et al., 2017). It has been suggested that AMPK activation indirectly, via protein synthesis inhibition, could preserve heart function by inhibiting ER stress and eventually prevent cardiomyocyte death.

#### 3.5.7 microRNA Expression

MicroRNAs are crucial regulators in the progression of cardiac hypertrophy. AMPK regulates many microRNAs, such as miR-195, miR-133a, and miR-451 during the course of cardiac hypertrophy. Among all, miR-133a has a key role against cardiac hypertrophy (Care et al., 2007). Adiponectin, a circulating adipose-derived cytokine could activate AMPK, elevates miR-133a level, and finally decreases cardiac hypertrophy induced by Ang II (Li et al., 2016). Some microRNAs are involved in AMPK pathway such as miR-195 (Chen et al., 2012) and miR-451 (Kuwabara et al., 2015), whose expression is elevated in hypertrophic cardiomyocytes, thereby inhibiting the activation of the AMPK/liver kinase B1 (LKB1) signaling axis and amplifying cardiac hypertrophy.

### 3.6 AMPK Genetic Mutations in Heart

So far, the only AMPK genetic mutations occurs in PRKAG2 (encoding the γ2 isoform of the nucleotide-binding subunit), leading to Wolff–Parkinson–White syndrome (WPWS), a type of heart disease (Arad et al., 2007). Ventricular pre-excitation (a premature excitation of the ventricles, detected by electrocardiogram) is a feature of this syndrome (Gollob et al., 2001). This condition is relatively rare, affecting 0.9–3% of the population (Rosner et al., 1999).

Heart is the preferential tissue where γ2 isoform is highly expressed (Cheung et al., 2000). In fact, the γ subunit mutation in WPWS leads to extra glycogen retention and accumulation in cardiomyocytes and these cells contribute to the accessory pathway formation (named the Bundle of Kent) leading to arrhythmias (Gollob 2003).

### 3.7 Diabetic Cardiomyopathy

One of the critical LV pathology that accompanies diabetes mellitus (DM), is diabetic cardiomyopathy. It can leads to HF if left untreated (Kannel et al., 1974). Studies have shown that AMPK displays anti-fibrotic effects against LV dysfunction in diabetic mouse model of both type 1 and type 2 DM. In db/db mice (a type 2 DM model), reduced AMPK activity, decreased contractile ability and inefficient cardiac metabolism has been described (Daniels 2010). In type 1 DM model (OVE26 mice), AMPK regulates autophagy (Xie et al., 2011). In comparison to wild type littermates, OVE26 mice presented LV dysfunction, lower AMPK function, and attenuated autophagy. Finally, ROS accumulation is a well known etiology for the development of diabetic cardiomyopathy (Seddon et al., 2007). In this regard, AMPK activation in cardiomyocytes prevents glucotoxicity by lowering NOX2-mediated ROS production (Balteau et al., 2014).

## 4 Natural AMPK Activators as Treatment of Cardiovascular Disorders

**FIGURE 1:**
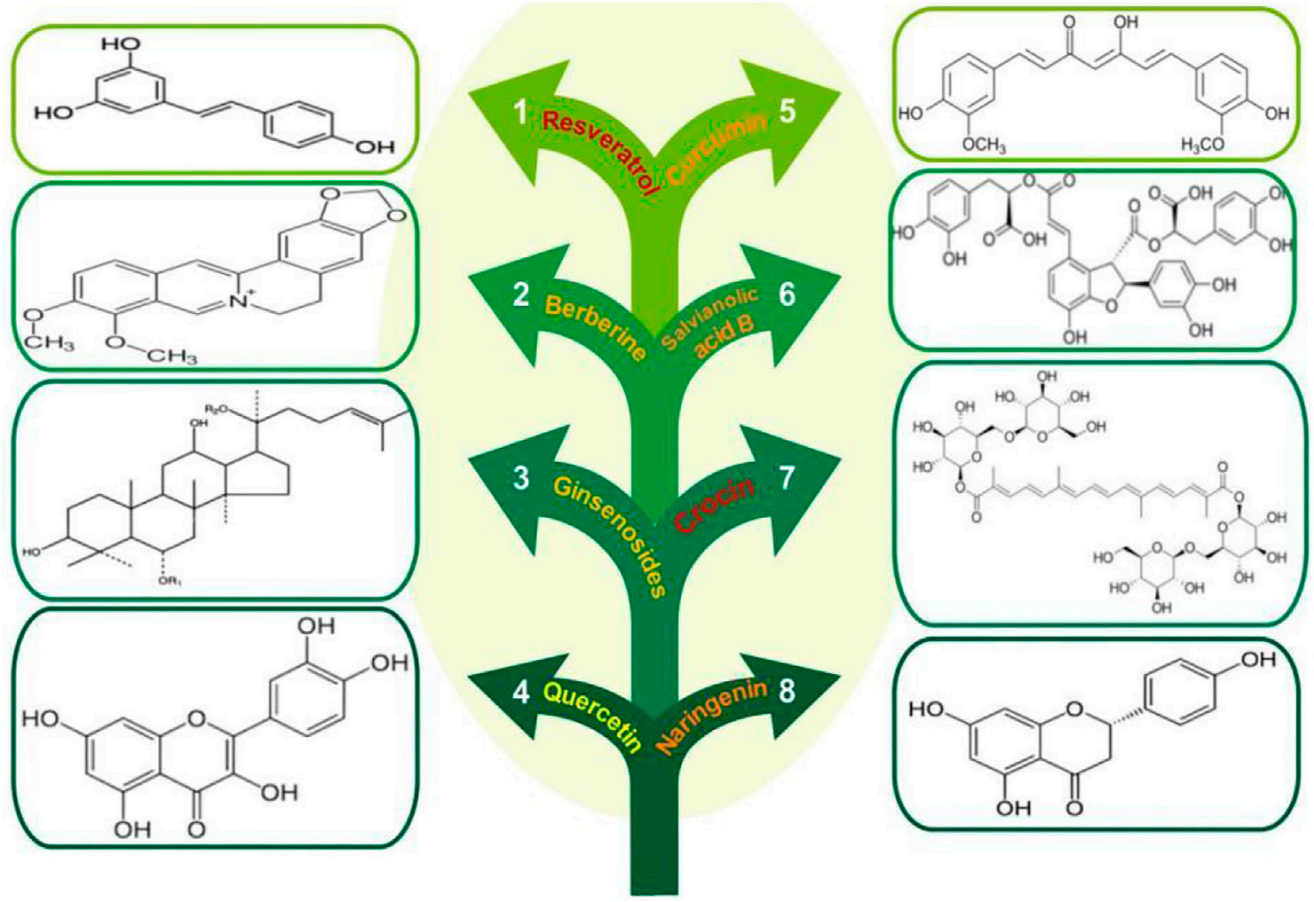
Chemical structures of the natural AMPK activators.

Based on the cardiovascular actions of AMPK, natural AMPK activators are of great therapeutic potential. A number of natural products and herbal constituents have been reported to activate AMPK and prevent CVD (summarized in Figure 1).

### 4.1 Resveratrol

Resveratrol (3,5,4′-trihydroxy-trans-stilbene), is a natural polyphenolic compound with three hydroxyl groups in its structure. It is widely distributed in edible fruits like berries, pomegranates, grape skin, peanuts, and many others. This polyphenol displays beneficial biological functions in the cardiovascular system due to its anti-inflammatory, anti-atherogenic, hypolipidemic, anti-platelet, and anti-oxidant properties (Cheng et al., 2020). It has been found that resveratrol through AMPK activation improved cardiac function and decreased the risk of CVD development. Several studies have indicated that natural AMPK activators can prevent the development of cardiac hypertrophy and resveratrol as a natural compound can inhibit hypertrophic growth. More recently, treatment with resveratrol has been demonstrated to reverse cardiac hypertrophy in mice following transverse aortic constriction surgery. Resveratrol attenuates phosphatase and tensin homolog (PTEN) degradation by inhibiting proteasome-dependent degradation, thereby leading to the inactivation of AKT/mTOR signaling pathway and activation of AMPK pathways (Chen et al., 2019).

One of the main therapeutic advantages of resveratrol is attributed to its effective antioxidant capacities. Oxidative stress has an important role in the development of hypertension. Resveratrol pretreatment decreases the harmful oxidative consequences of hypertension and cardiac hypertrophy. To test the effect of resveratrol on hypertension, fructose-fed rats were treated with resveratrol (10 mg/kg). Results indicated that resveratrol considerably lowered blood pressure and ROS production, enhanced nitric oxide levels by activating AMPK in the fructose-induced hypertensive rat model (Cheng et al., 2014). Another study in animal models of hypertrophic cardiomyopathy also demonstrated the positive role of resveratrol via increasing the activation of the hierarchical LKB1/AMPK signaling pathway. Blood and cardiac levels of lipid peroxidation byproduct 4-hydroxy-2-nonenal (HNE) is increased during hypertension. HNE inhibits the activation of LKB1 and subsequent AMPK, consistent with this inhibition, mTOR and p70S6 kinase are activated. Resveratrol has been shown to reduce cardiac HNE levels and provide beneficial effects on LV hypertrophy (Dolinsky et al., 2009). According to another study (Dolinsky et al., 2013), Dolinsky et al. showed that resveratrol reduced HNE levels, activated eNOS, and reduced p70S6K activity by activating LKB1 and AMPK in angiotensin II (AngII) -induced hypertensive mice and rats. Resveratrol activates AMPK and blocks the Akt/mTOR/p70S6K pathways which lead to the reduction of smooth muscle cell proliferation and DNA synthesis (Brito et al., 2009).

Researchers have noted that acute treatment with resveratrol (100 μmol/L) can reduce cell growth in rat cardiac myocytes by increasing AMPK and ACC phosphorylation as well as inhibiting Akt activation in the hypertrophic process (Chan et al., 2008). It was observed that resveratrol (30 μmol/L) significantly prevented increases in cardiomyocyte size and protein synthesis in norepinephrine-induced cardiac hypertrophy in adult rats. Moreover, AMPK activation conferred by resveratrol increased the level of nitric oxide (Thandapilly et al., 2011). There was a significant reduction in blood pressure, Ras homolog gene family member A (RhoA) activity and levels of phosphorylated myosin phosphatase-targeting subunit 1 (MYPT1), and myosin light chain (MLC) by resveratrol in AngII-treated mice (Cao et al., 2014).

In a cellular study performed by Hwang *et al.*, potential anti-apoptotic effect of resveratrol (50, 100 μmol/L) was assessed on H9c2 cardiac muscle cells. Results of this investigation suggested that resveratrol acts as a powerful AMPK activator against H_2_O_2_-induced cell death and apoptosis (Hwang et al., 2008). AMPK is a pivotal enzyme associated with a wide range of cardiac metabolic pathways such as protein synthesis, glucose metabolism, and fatty acid oxidation, as well as insulin-sensitization. There is also some evidence suggesting the efficacy of resveratrol therapy in diabetic cardiomyopathy. AMPK activation by resveratrol prevented hyperglycemia-induced apoptosis and oxidative stress, by inhibiting NADPH oxidase-derived ROS production in cardiomyocytes (Guo et al., 2015). In addition, in a mouse model of type 2 diabetes mellitus, resveratrol (10 mg/kg) via AMPK activation can decrease disulfide bond A oxidoreductase-like protein (DsbA-L) and adiponectin (APN) levels which are crucial for the prevention of myocardial ischemia/reperfusion injury in diabetes (Yang et al., 2016).

It has been demonstrated that resveratrol (10–100 μmol/L) prevented endothelial dysfunction related to hyperglycemia in HUVECs. Activation of AMPK by resveratrol increased eNOS activity and NO production, also improved vascular function and glycemic control (Xu et al., 2009). Kanamori et al. (2013) have reported that resveratrol (50 mg/kg/day) reduced cardiomyocyte size, heart weight/body weight ratio, promoted autophagy by activating AMPK in postinfarcted LV. AMPK plays a major role in the prevention of HF and cardiac dysfunction. Resveratrol-enriched diet feeding increased SIRT1 expression and AMPK activation, which lead to improved cardiac function in HF (Gu et al., 2014).

### 4.2 Berberine

Berberine is an eminent component of traditional Chinese medicine. Many studies revealed the cardioprotective effects of berberine is dependent on AMPK activation (Feng et al, 2019). It is suggested that ER stress is the main cause of endothelial dysfunction during hypertension. Berberine (1 μmol/L) significantly suppressed ER stress by activating AMPK in spontaneously hypertensive rats. Moreover, evidence showed that endothelium-dependent contractions are reduced by increasing AMPK phosphorylation in arteries (Liu et al., 2015). The results demonstrated that berberine attenuated Ang II-induced myocardial hypertrophy by regulating LC3 protein and AMPK phosphorylation *in vitro* conditions (Zeng et al., 2019). The beneficial effects of berberine (0.25–4.0 μmol/L) on mitochondrial dysfunction are suggested to be the result of suppressing doxorubicin-induced cardiac injury via increasing AMPK and reducing AMP/ATP ratio, as well as promoting Bcl-2 protein level (Lv et al., 2012). In line with this observation, AMPK activation by berberine leads to inhibited autophagy and apoptosis in hypoxia-induced myocardial cell injury (Jia et al., 2017).

It has been well established that berberine attenuates vascular inflammation and leads to the suppression of atherogenesis. Treatment with berberine has been found to reduce oxidative stress and atherosclerosis in ApoE^−/−^ mice followed by AMPK-dependent expression of upregulation of uncoupling protein 2 (UCP2) (Wang et al., 2011). In another series of experiments, berberine (5, 10, 20 mg/kg/d) suppressed the formation and accumulation of foam cells by activating the AMPK, SIRT1, and PPAR-γ signaling pathways (Chi et al., 2014). In a recently published study, it has been reported that berberine significantly improved endothelial dysfunction caused by low shear stress-via increasing AMPK phosphorylation. In fact, AMPK inhibited hyaluronidase 2 (Hyal2) pathway and p47^phox^ phosphorylation, also modulated hyaluronic acid synthase 2 (HAS2) activity in HUVECs model (Yang et al., 2019). A further study on endothelial dysfunction has indicated that AMPK activation by berberine increases eNOS activity and decreases the protein expression of NADPH oxidase 4 (NOX4), which elevates NO levels in vascular endothelial cells (Zhang et al., 2013). The ability of berberine in the inhibition of platelet-derived growth factor (PDGF)-induced VSMCs proliferation through activation of AMPK/p53/p21^Cip1^ pathway has also been demonstrated *in vitro* (Liang et al., 2008). Berberine-induced activation of AMPK improves cardiac fibrosis, an effect that has been linked to the inhibition of mTOR/p70S6K signaling pathway (Ai et al., 2015).

Current evidence suggests berberine is a widely-used AMPK activator for the treatment of type 2 diabetes with or without CVD (Feng et al., 2019). Berberine treatment for 7 days in insulin-resistant rats (by high fat diet feeding) increased AMP/ATP and ADP/ATP ratios, AKT phosphorylation, and decreased glycogen synthase kinase 3β (GSK3β) through AMPK activation in ischemia–reperfused diabetic rat hearts. This suggests that berberine can be used as a cardioprotective agent against ischemia-reperfusion injury related to diabetes (Chang et al., 2016). In diabetic cardiomyopathic rats, berberine attenuated cardiac hypertrophy through activating both AMPK and AKT activity, and inhibited the expression of GSK3β (Chang et al., 2015). Another important effect of berberine is its ability to promote glucose uptake and glucose consumption in normal or insulin-resistant H9c2 cardiomyocyte cells via AMPK activation (Chang et al., 2013). Berberine (100 nmol/L) attenuated high glucose-induced (50 mmol/L) hypertrophy, decreased LC3-II level, inhibited mitochondrial fission and mTOR, and restored autophagic flux in H9c2 cells by targeting AMPK (Hang et al., 2018).

### 4.3 Ginsenosides

Ginsenosides, the main bioactive ingredients of ginseng, are a group of saponin compounds with a wide range of biological and therapeutic activities. Ginsenosides (Rg1, Rb1, Rd, Re, and Rg3) are the representative ginsenosides that are most frequently studied. The anti-apoptotic and anti-atherosclerotic effects of Rg1 were investigated *in vitro*. The levels of autophagy-related proteins such as Atg5, LC3, Beclin1, and p62/SQSMT1 were increased by Rg1 (50 μmol/L). The result demonstrated that Rg1 could suppress apoptosis and promote autophagic flux in macrophages by activation of AMPK/mTOR signaling pathway (Yang et al., 2018). Rg1 (20 mg/kg) is also able to alleviate inflammation and cardiac oxidative stress in diabetic rats, through promoting AMPK/Nrf2/HO-1 signaling pathway (Qin et al., 2019).

Recent findings by Zheng et al. (2020) have shown that Rb1 could inhibit H_2_O_2_-induced endothelial dysfunction and increased SIRT1, eNOS, NO production, and suppressed expression of plasminogen activator inhibitor-1 (PAI-1). AMPK activation was also necessary for Rb1 to reduce endothelial aging in this study. Rb1 could ameliorate the autophagy of cardiomyocytes by up-regulating AMPK pathway and reducing levels of p62 and cathepsin B in rat cardiomyocytes under hypoxia conditions (Dai et al., 2019). Sun *et al.* (Sun et al., 2020) assessed the molecular mechanisms by which Rg3 exerts myocardial protection. The following benefits were observed: promotion of ACC phosphorylation, enhancemeng of autophagy in isoproterenol-induced myocardial infarction (MI), and inhibition of myocardium apoptosis. This myocardial protective effects have also been observed in Re (135 mg/kg) treated rats, in which Re significantly improved cardiac function and LV fibrosis in rat MI model by regulating AMPK/TGF-β1/Smad2/3 signaling (Yu et al., 2020). Hyperlipidemia is considered to be one of the important risk factors for coronary heart disease, such as atherosclerosis, MI, heart attacks. The beneficial and protective effects of Rg3 have been related to the decrease of intracellular cholesterol and triglyceride levels (Lee et al., 2012).

### 4.4 Quercetin

Quercetin, is a flavonoid that has been isolated from a variety of vegetables and fruits including citrus, berries, apples, and tea. There have been numerous studies suggesting that quercetin exhibits anti-inflammatory, antioxidative, cardioprotective, and endothelial protective effects (Chekalina et al., 2018). It has been shown that quercetin (5 and 10 µM) was able to activate AMPK, eNOS, and promote NO production, leading to improved vascular function in cellular model of endothelial dysfunction (Shen et al., 2012). In VSMCs, Kim *et al.* found that the phosphorylation of AMPK and LKB1 by quercetin (25, 50, 100 µM) regulated the expression of MLC kinase and the phosphorylated myosin light chain (*p*-MLC), as well as inhibited phenylephrine-induced vasoconstriction (Kim et al., 2018).

### 4.5 Curcumin

Curcumin is a natural polyphenol found in turmeric, which displays beneficial functions including anti-oxidant, anti-inflammatory, anti-angiogenic, anti-thrombotic effects dependent on AMPK activation (Li et al., 2020; Singh et al., 2021). In one study, *in vivo* and *in vitro* data indicated that curcumin (200 mg/kg) significantly improved cardiac apoptosis in the hearts of diabetic mice. Activation AMPK and JNK1 by curcumin regulated autophagy and alleviated apoptosis through phosphorylating Bim and Bcl-2 proteins, which lead to the disruption of their interactions with Beclin1 (Yao et al., 2018). A recent study has presented that curcumin-loaded nanoparticles attenuated oxidative stress and inhibited the intracellular ROS increase via AMPK activation in palmitate-induced cardiomyocyte apoptosis (Zhang et al., 2019). Treatment with curcumin (25 μmol/L) suppressed type I and type III collagen synthesis and TGF-β1 production in cultured cardiac fibroblasts, as well as inhibited cardiac fibrosis. Interestingly, one-month administration of dietary curcumin in aged rats restored cerebrovascular endothelium-dependent vasorelaxation. Curcumin also improved AMPK phosphorylation and attenuated ROS production in cultured endothelial cells and cerebral arteries of aged animals. However, after AMPK inhibition, the beneficiary effects of curcumin were not observed, indicating AMPK dependency (Pu et al., 2013). Despite other findings showing that AMPK activation is effective to prevent cardiac fibrosis, Guo *et al.* reported that curcumin inhibited AMPK/p38 MAPK activity in their study results (Guo et al., 2018), which warrants further study.

### 4.6 Salvianolic Acid B

Salvianolic acid B is a natural antioxidant compound that is found in *Salvia miltiorrhiza (Danshen)* roots (Fang et al., 2018; Li et al., 2018). This phenolic acid has been traditionally used for the treatment of CVD. It was found that, in the cultured endothelial cells, treatment of myocardial infarction with salvianolic acid B produced an increase in NO production and activated phosphorylation of Akt and AMPK. Additionally, salvianolic acid B increased cationic amino acid transporters and eNOS expression through the AMPK/PI3K/Akt pathway, leading to stimulated L-arginine uptake (Pan et al., 2011). It has been demonstrated that salvianolic acid B (5, 10, 20 μg/ml) protected HUVECs against H_2_O_2_-induced apoptosis via promoting autophagy by AMPK activation and mTOR inhibition (Gao et al., 2019). Yang et al. (2019) suggested that salvianolic acid B can suppress apoptosis and increase cell viability in oxygen-glucose deprivation injury in H9c2 cells through regulating murine double minute 2 (MDM2)/p53 and AMPK signaling pathways.

### 4.7 Crocin

Crocin, the major bioactive phytochemical component of saffron, can be used as a novel therapeutic agent against CVD. The evidence suggests that crocin pretreatment can promote autophagy during ischemia and reperfusion injury, and reduced myocardial apoptosis, infarct size, and necrosis, accompanied by the activation of AMPK (Zeng et al., 2016). The effect of crocin (10, 20 mg/kg) on diabetic heart dysfunction was evaluated in streptozotocin-induced diabetic rats. Treatment of crocin resulted in a reduction of myocardial apoptosis, as evidenced by increased phosphorylation of myocardial AMPK, normalized levels of autophagy marker proteins, and improved cardiac function in diabetic animals. Therefore, crocin may be a potential therapeutic natural AMPK activator for diabetic cardiomyopathy treatment (Feidantsis et al., 2018).

### 4.8 Naringenin

Naringenin, a naturally-occurring plant flavonoid, is abundantly present in common vegetables and fruits. It is recognized as an effective treatment for CVD (Heidary Moghaddam et al., 2020). Saenz et al. (2018) have been reported that naringenin can prevent foam cell progression and atherosclerosis in human macrophages models. Naringenin treatment (100 µM) increased cholesterol efflux and suppressed macrophage migration *via* AMPK activation. Due to obvious AMPK and SIRT3 activation capacity, the cardiovascular protective effects of naringenin could involve both targets. A study has shown that naringenin reduces cardiac damage in animal models by activating AMPK-SIRT3 signaling pathway, suggesting that naringenin can be exploited as an effective therapeutic agent for ischemic heart disease (Yu et al., 2019).

## 5 Natural AMPK Activators in Cardiovascular Outcome

In the prior section, the cardioprotective effects of natural AMPK activators are presented. Most of these natural cardioprotective agents have only been evaluated in animal models and *in vitro* studies. Some of these compounds have entered clinical trials and have demonstrated positive results. In this section, research studies involving the clinical trials of natural AMPK activators are discussed.

In one study, a total of 84 male and female patients with coronary artery disease were used for evaluating the beneficial effects of crocin. Patients were randomly divided into three groups: group 1 received a crocin capsule of 30 mg/day daily; group 2 received a saffron aqueous extract capsule of 30 mg/day daily and group 3 received placebo capsules with similar shapes for 4 weeks. The comparison between groups revealed that there were significantly enhanced expression/activity of SIRT1 and AMPK, also decreased expression of LOX1 and NF-κB in the crocin treated group, compared with the placebo group. In addition, crocin could also reduce serum levels of oxidized low-density lipoprotein and monocyte chemoattractant protein 1 (MCP-1) in these patients. Crocin-treated group also had significant higher efficacy than saffron aqueous extract-treated group (Abedimanesh et al., 2020). Another clinical trial has been launched to evaluate the effectiveness of berberine on galectin-3 expression and macrophage activation in patients with acute coronary syndrome. Galectin-3, as a disease-relevant biomarker, is increased in atherosclerotic lesions and inflammation. In a single-blinded trial, 45 patients were randomly divided into two groups according to 2:1 randomization ratio. One group consisted of 30 patients treated with 300 mg of berberine hydrochloride for 3 months, and the other group consisted of 15 patients who had received only standard therapy. The results indicated that berberine reduces oxidized low-density lipoprotein-induced macrophage activation via decreasing galectin-3 expression by activating AMPK signaling and suppressing NF-κB pathway (Pei et al., 2019).

Berberine has been used to protect cardiomyoblast cells from apoptosis as a preventive and curative treatment of myocardial injury in postoperative patients. The results evidenced that berberine provided a reduction of all inflammatory biomarkers after operation in patients with acute myocardial infarction, as well as inhibiting autophagy and apoptosis in H9C2 cells through the AMPK/mTOR pathway (Qing et al., 2018).

The clinical benefits of resveratrol have been investigated in patients with hypertension and dyslipidemia. Researchers observed that resveratrol leads to reduced endothelial dysfunction by modulation of NO bioavailability in diseased human vessels. The level of tetrahydrobiopterin (BH4) increased after resveratrol treatment, suggesting resveratrol’s effects to increase AMPK and eNOS activation, as well as attenuation of vascular oxidative stress (Carrizzo et al., 2013).

## 6 Conclusion

**FIGURE 2:**
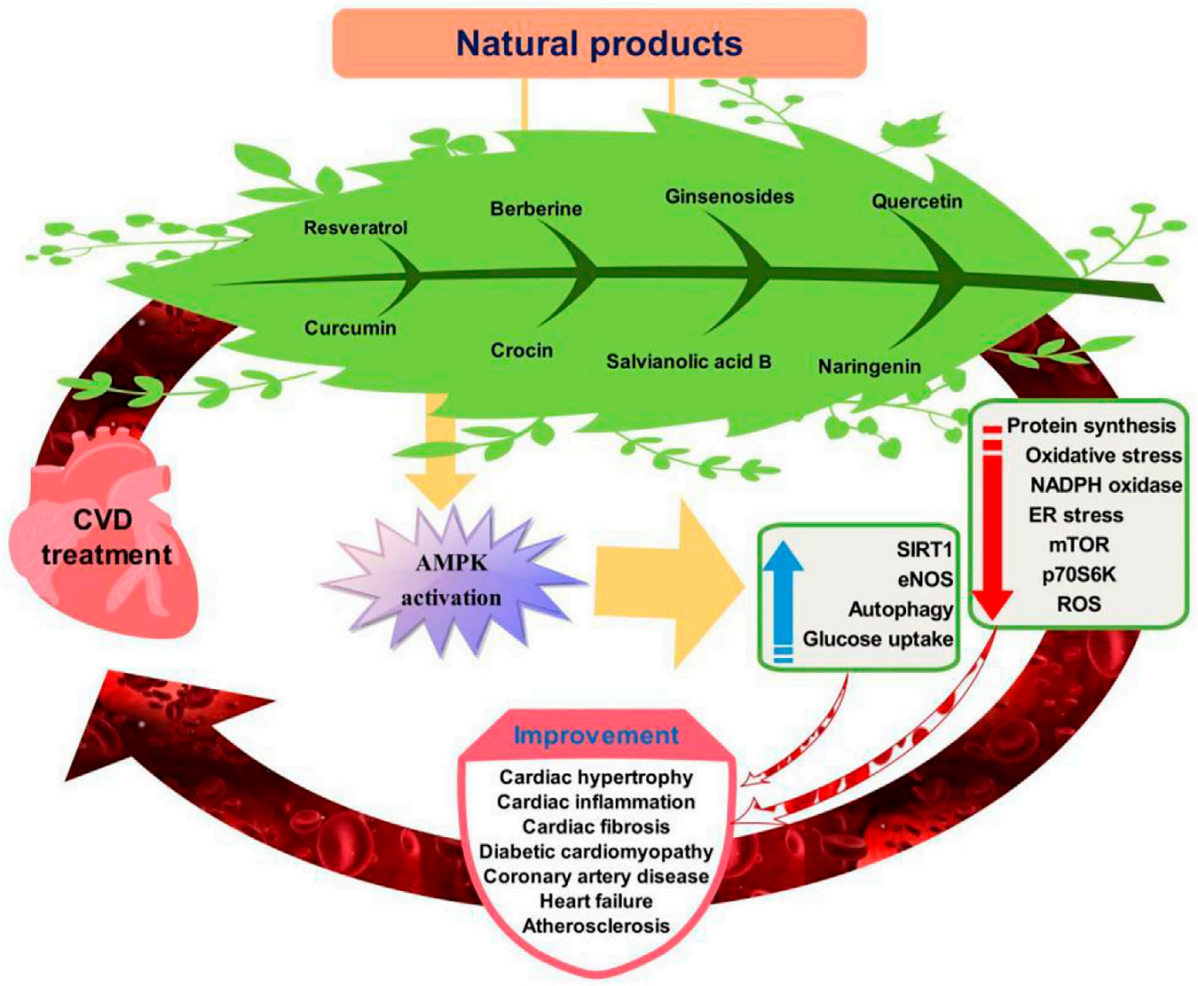
Targeting of AMPK signaling pathway by natural products for cardiovascular disease prevention.

AMPK is the fuel sensor and key target of cardiometabolic homeostasis and diseases. Emerging evidence has suggested AMPK activators from naturally-occuring sources provide notable cardiovascular benefits (Figure 2). AMPK as a sensor of cellular energy also regulate cardiac system bioenergetics and energy metabolism. AMPK activation prevents cardiometabolic disease by its capacity to lower blood pressure, glucolipids, ROS production, and improve NO bioavailability. AMPK activators thus serve as novel potential drugs in gatekeeping cardiovascular health and preventing cardiovascular disease.

However, translational validity regarding the association with natural products, AMPK and CVD tends to be weak. These correlations do not necessarily modify clinical events nor directly address “prevention” in terms of superphysiological or pharmacological doses are used in rodent models or cultured cells. The calculation of human doses in rodents and vice versa is also a critical factor. More research is needed to clarify the exact mechanism of action associated with AMPK activation by natural products as well as to explore the safety and efficacy of natural AMPK activators to treat patients with CVD at therapeutically relevant or human equivalent concentrations and doses.
